# How Readable Is the Information the United Kingdom's Statutory Health and Social Care Professional Regulators Provide for the Public to Engage With Fitness to Practise Processes?

**DOI:** 10.1111/hex.70067

**Published:** 2024-10-16

**Authors:** Sharif Haider, Louise M. Wallace

**Affiliations:** ^1^ Health and Social Care The Open University Milton Keynes UK; ^2^ Faculty of Well‐Being, Education & Language Studies The Open University Milton Keynes UK

**Keywords:** fitness to practise (FtP), healthcare professionals, professional regulation, readability, regulatory bodies, social care professionals

## Abstract

**Background:**

The public are an important source of notifications and evidence for the investigation of concerns by regulators of professionals. The website is an important source of information for the public, but the complexity of information presented to engage with the public is unknown.

**Objectives:**

This study explored the readability of information provided for the public to engage with fitness to practise processes by examining the websites of the 13 UK statutory health and social care professional regulators.

**Methods:**

Six readability algorithms were utilised to calculate the readability scores of 180 general and 8 easy‐read documents published for the 15 sites of the United Kingdom's 13 health and social care statutory professional regulatory bodies. These tests were the Flesch Kincaid Reading Ease, the Flesch Kincaid Grade Level, the Gunning Fog Score, the Simple Measure of Gobbledygook (SMOG) Index, the Coleman Liau Index and the Automated Readability Index (ARI).

**Results:**

All the fitness to practise documents analysed in this study are written at a level too difficult for most of the general population to read, except one easy‐read document. There was also considerable variation in readability across resources for the same regulator, which could be confusing. Regulatory bodies risk excluding a large proportion of UK adults who may want to engage with professional regulatory proceedings.

**Conclusions:**

This is the first comparative analysis of readability conducted independent of the regulators of the fitness to practise website documents of health and social care regulators. The public are a key source of evidence in regulatory proceedings. Regulators could improve public engagement by addressing the complexity of language used.

**Public Contribution:**

Our advisory group of people with lived experience of involvement as members of the public in fitness to practise proceedings discussed the findings and contributed to the recommendations.

## 1 Introduction

When a patient, service user or their family believe that they have experienced poor or harmful care and associate this with specific practitioners, they may complain to the employing organisations. In a different process, they may also raise a ‘concern’ with the professional regulator of the practitioner, although not all practitioners are required to be regulated in the United Kingdom. There are 13 statutory health and social care regulators in the United Kingdom, and others are voluntarily registered with accredited registers. These bodies promote standards of practice specific to the professions. They hold registers of practitioners. If a concern is raised about a professional's conduct, competence or that their health is adversely impacting their practice, the regulator is required by law to ascertain if the person is a registrant, to find out if the allegations meet a threshold of seriousness requiring further action, including whether there is a case to answer. The regulator undertakes an investigation, and the registrant is informed and generally required to respond to the evidence presented. This evidence may be tested in a tribunal hearing before a panel of adjudicators appointed by, but independent of, the regulator. The patient, service user or their family or others who observed the events relevant to the standards of practice that may have been breached may be called to give evidence at any stage, including at the hearing. Although many outcomes are possible, the most severe is removal from the register, often known as ‘striking off’. In the United Kingdom, these regulatory procedures are known as the fitness to practise (FtP) process.

The public raise a substantial proportion of concerns to UK regulators. For example, 70% of new concerns in the 40 months since the inception of Social Work England in December 2019 (the regulator of social workers in England) were from the public (personal communication, Sharif Haider, October 11, 2024), 74.7% for the General Medical Council [1] and 38% in the most recent year for nurses and midwives [2]. The website of the regulatory bodies may be the first source of information for the public in determining what they should do if they are dissatisfied with the registrant's practice. It makes no difference whether it has been investigated by the employer or not; the public can expect to learn how to raise a concern and what to expect afterwards from regulatory bodies' websites. For regulators to provide information that enables the public to raise a concern and to give their evidence, it is important to understand the complexity of information that regulators provide on their websites.

There is no published research on the complexity of the regulators' websites in the United Kingdom or elsewhere, but we draw on what is known about the public's use of online information related to handling complaints, where it has been found that effective information is crucial for patient understanding [3]. Mattarozzi et al. found that patients often complain about receiving information inconsistent with the truth, which can stem from misunderstandings or unmet expectations. They suggest using language that patients can easily understand [4]. Similarly, a realist review of complaints in health services by Dael et al. suggests that providing clear and accessible information enhances patients' understanding and engagement, consequently improving health literacy and the quality of care [5]. The term health literacy was coined in the 1970s and refers to an individual's ability to use general literacy skills, that is, reading, speaking, listening, writing and numeracy in obtaining appraising, understanding, synthesising, communicating and applying health‐related information [6]. It is regarded as a significant single predictor of an individual's health state; insufficient health literacy may result in increased visits to accident and emergency departments and increased healthcare costs. The creation of accessible and readable health information is a requirement for adequate health literacy; consequently, readability (discussed below) and comprehension are key characteristics of health literacy [4].

Previous health education research shows that online information related to health plays a significant role in patients making decisions about their health [3]. Readability is crucial, as it directly impacts individuals' ability to understand and utilise health information effectively and empowers individuals to make informed health decisions. High readability ensures that health materials are accessible to a broader audience, particularly, those with varying levels of education and literacy skills. Individuals with low health literacy face higher risks of adverse health outcomes and lower participation in preventive activities. Furthermore, the readability of online health resources is often compromised by complex medical terminology and poor webpage design, which can deter patients from seeking necessary medical procedures [4].

Readability matters because it is an indicator of the complexity of language and the proportion of adults able to easily understand text. The average reading level in the United Kingdom is estimated to be year 5; in other words, the average reading age of adults in the United Kingdom is typical of a child in UK education at 9 years old [7, 8, 9], but research evidence suggested that some have a much lower reading level [10, 11, 12]. Health Education England suggested that it is acceptable if health information is written at a level that can be understood by 11‐year‐old school children [13]; similarly, the American Medical Association has recommended to write materials that can be understood by 11‐ to 12‐year‐old children [14].

We sought to ascertain the reading ages required to understand the public‐facing information about FtP by the 13 UK statutory health and social care regulators, and as there are no similar studies globally, it will therefore have relevance beyond the United Kingdom.

### 1.1 Policy Context and Readability

It is recognised that the documents published by the UK government are too complex for at least half of the readers, which is also the case with other private and public organisations [15]. Rowlands et al. [16] found that 43% of written health information is too complex for UK adults to understand. They found that 61% could not understand the text if statistical information was included. As a result, the NHS Information Standard stated that health literacy, including accessibility and readability, should be considered when developing material for a specific audience [17]. This provides a context that suggests that UK regulators should be designing information to similar standards of readability as expected of those providing health information.

Unlike the United States, the United Kingdom does not have a legal framework for using plain English in official documents but the Plain English Language Campaign in the UK advocates against gobbledygook, jargon and misleading public information [18]. They accredit documents, website materials and apps with a Crystal Mark Seal of excellence that shows that the materials reached their high standards and are clear, well‐designed and accessible. It is constantly highlighted that most of the documents produced by the UK government are too complex for most people to understand. There are too many long sentences and include technical and management jargon and acronyms [19]. An independent language consultancy, Linguistic Landscapes, analysed 6 years of research into the UK Department of Health documents and found that readers scored all the documents as difficult to understand and boring [19].

Easy‐read documents are intended primarily for people with learning difficulties for their special literacy needs, and is the term typically used in the United Kingdom, although alternatives are ‘easy to read’ or ‘easier information’. These typically include short sentences and pictograms and commonly seen symbols such as road signs. The use of ‘easy‐read’ documents by organisations may assist them in complying with the UK Equality Act (2010), as it may demonstrate that the organisation is offering equitable access to individuals with disabilities. Organisations also need to comply with the United Nations Convention on the Rights of Persons with Disabilities (UNCRPD) by making their documents easily accessible to individuals with disabilities. The Inclusion Europe [20] international campaigning organisation, which is supported by the European Commission, developed five standards for making information easy to read and understand based on four different formats of producing and sharing information, with general standards for easy‐to‐understand information and specific standards for written, electronic, video and audio information. The goal of easy‐to‐read documents is to provide and explain information in a simple and straightforward manner so that people can readily understand the contents. Chinn and Homeyeard [21] found in their systematic review that this type of publication gives knowledge about regulations and advice in an accessible manner, allowing persons with learning disabilities to become self‐determined and empowered. By doing so, organisations using easy‐read documents aim to reduce health disparities and promote active citizenship. They assert that if information is not supplied in easy‐to‐read forms, some people may struggle not only to understand the contents of the document but also to make essential health and social care decisions. In this study, we examine regulators' easy‐read documents for readability.

There are other advantages to using simple written language. People with the highest literacy levels and the most knowledge on a subject frequently prefer documents presented in plain English [19]. Trudeau [22] reported that when given the option to read phrases written in simple English, 80% of participants opted to do so. This preference is stronger when the topic matter is difficult. According to MORI [23] research, persons with the most experience and high reading levels used less time to read dry and difficult prose. As a result, the Department of Health in the UK advised writing short sentences with clear and simple terms and avoiding jargon [19].

To determine how well a target population of adult readers may engage with a text, readability tests have been used for decades in health promotion research [24, 25]. Readability is about the level of competence required to understand and grasp a given text [2, 25, 26]. According to Dale and Chall [27], readability is the ‘sum total (including the interactions) of all those elements within a given piece of printed materials that affects the success a group of readers have with it’ (p. 23). Simply, it can be defined as ‘the level of knowledge and skill required to make full sense of a given printed text’ [26]. In other words, ‘the ease of understanding or comprehension due to writing style’ [28].

Readability is an important indicator of the proportion of the adult population who can engage with a text because of their literacy. In 2015, OECD conducted a survey on adult reading skills, the Programme for the International Assessment of Adult Competencies, and found that 16.4% of adults (7.7 m people) in England and 17.4% of adults (6000 people) in Northern Ireland have literacy levels at or below level 1 [29]. This proportion will have very poor literacy skills, and they can read brief texts on familiar topics; they have basic vocabulary knowledge [28]. Literacy for large sections of the adult population in other parts of the United Kingdom are similarly poor [29, 30].

### 1.2 Research Question

How readable is the information that the 13 UK statutory health and social care professional regulators provide for the public to engage with FtP processes?

## 2 Materials and Methods

The average reading level in the United Kingdom is estimated to be year 5; in other words, the average reading age of adults in the United Kingdom is typical of a child in UK education at 9 years old [7, 8, 9], but research evidence suggested that some have a much lower reading level [10, 11, 12].

There are a number of formulas available to examine readability; they provide a quantitative way to estimate the reading difficulty as well as predict how readable a document is for the target audience. There are about 200 formulas available to calculate how easy a text is to read and understand. Most often, two variables are measured by each of these formulas: (i) word length or the number of syllables and (ii) syntactic complexity, which can be correlated with sentence length [31], and sentence length correlates with comprehension [32, 33]. These formulas do not assess readers' prior knowledge about the subject, their motivation and comprehension. They also do not take into account the familiarity of words. However, they are useful because they can give an indication of the likely level of literacy required to understand the regulators' resources.

Higher readability scores suggest an easier read overall, whereas lower values indicate a more challenging read. A readability score of 85%, for example, is similar to school‐age education for 9‐year‐olds (Table 1), whereas a score of 50% indicates that adults who studied to 18 years old would easily understand these materials. Furthermore, a score of 70% is a desirable target according to the National Institute of Health and the American Medical Association, as their recommendation is to write text to be readable by adults with literacy at least at sixth grade in the United States, which is equivalent to 11‐ to 12‐year‐old school children in the United Kingdom [34]. Similarly, according to Health Education England, patient information should be written at the year 7 level, which is equivalent to the literacy expected of an 11‐ to 12‐year‐old [13].

**Table 1:** How readability scores are associated with reading age of school children educated in the United Kingdom.

Reading age	UK school year	Scores
5–10	Years 1–6	85%+
11–13	Years 7–9	70%+
14–18	Years 10–13	50%+

*Note:* Readability scores and their associated reading age based on WebFX scores (https://originality.ai/blog/webfx-readability-test-tool-review) [25, 35].

There is no consensus between experts about the best readability formula for online materials aimed for the general public [36]. Therefore, as recommended by a number of researchers [25, 35], several readability formulas are used in combination. This study utilised a variety of readability formulas: the Flesch Kincaid Reading Ease, the Flesch Kincaid Grade Level, the Gunning Fog Score, the Simple Measure of Gobbledygook (SMOG) Index, the Coleman Liau Index and the Automated Readability Index (ARI). Both Coleman Liau and ARI rely on counting characters, words and sentences. The other indices consider the number of syllables and complex words (polysyllabics—with 3 or more syllables) too. By using these formulas, it is possible to understand the language level and complexity of the text published on the websites. As computer programs are used to calculate readability, the selection validity threats posed by humans who may unintentionally introduce bias in their selection of text will be eliminated [37].

Six readability tests were carried out by using computer software on selected documents published for the 15 sites of the United Kingdom's 13 health and social care statutory professional regulatory bodies. The tribunal services of two regulators are separate entities to the main website and were included as separate websites. Microsoft excel program was applied to capture all data.

### 2.1 Resources Included

The websites were searched for web pages and downloadable documents in March 2022. Documents were then included and downloaded for analysis if they had content aimed at the public (patients and service users) and related to raising a concern, the subsequent investigation process, giving a witness statement, attending a hearing and being cross‐examined and support for the involvement in the FtP process. Videos were not included unless they had a transcript. Resources aimed at employers or registrants alone were not included. The text of easy‐read documents was included.

A program called WebFX was used to combine all the formulas to assess readability. Documents were uploaded to WebFX software, and the program produced the readability score for each document. Then, the mean scores of all the documents were calculated. Only available online documents related to FtP were uploaded to the software.

## 3 Results

The number of documents included by regulator were General Chiropractic Council (GCC) (*n* = 5), General Dental Council (GDC) (*n* = 18), General Medical Council (GMC) (*n* = 32), General Optical Council (GOC) (*n* = 12), General Osteopathic Council (GosC) (*n* = 5), General Pharmaceutical Council (GPhC) (*n* = 15), Health and Care Professions Council (HCPC) (*n* = 12), Health and Care Practitioners Tribunal Service (HCPTS) *n* = 11), Medical Practitioners Tribunal Service (MPTS) (*n* = 10), Northern Ireland Social Care Council (NISCC) (*n* = 7), the Pharmaceutical Society of Northern Ireland (PSNI) (*n* = 4), Nursing and Midwifery Council (NMC) (*n* = 33), Social Care Wales (SCW) (*n* = 3), Scottish Social Services Council (SSSC) (*n* = 7) and Social work England (SWE) (*n* = 14).

There were 188 documents in total. The number of documents varied from 3 to 33 between regulators, a greater than tenfold difference.

**Figure 1:**
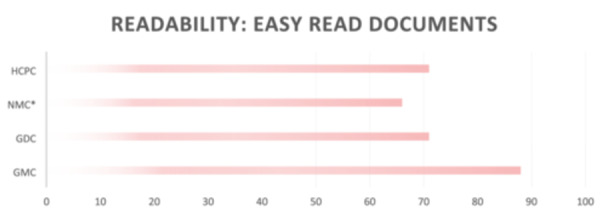
Readability scores for easy‐read documents. *The score for NMS is based on 5 documents.

Given this variability, we examined the means and ranges of readability scores of 180 documents for each regulator and tribunal service (see Table 2), and separately, the 8 easy‐read documents in Figure 1. Readability tests were applied across the suite of FtP documents for each regulator and show that there is a wide range of scores of documents within each regulatory body, meaning that the reading level varies within the regulatory body as well as across regulatory bodies.

**Table 2:** Comparison of highest (requires lower literacy) and lowest (requires higher literacy) scores on readability tests and the mean across general documents by regulator (*n* = 180).

Regulator	Highest scores	Lowest scores	Average scores	Standard deviation	Confidence interval (95%)
GCC	60	24	43	13.5	(59.2–25.8)
GDC	58	28	50	7.6	(44.9–52.7)
GMC	71	38	60	9.4	(55.6–62.56)
GOC	62	27	47	11	(55.6–62.5)
GOsC	59	39	50	8	(59.4–39.8)
GPhc	64	31	50	12.2	(43.2–56.8)
HCPC	55	19	46	11	(36.6–51)
HCPTS	63	48	56	5.5	(52.2–59.6)
MPTS	78	45	64	9.3	(57–70.4)
NISCC	68	35	51	12	(48.3–54.2)
NMC	65	25	53	7.6	(48.3–54.2)
PSNI	44	30	40	6.4	(29.3–49.9)
SCW	73	53	58	13.3	(24.5–90.9)
SSSC	66	47	60	5.9	(54.2–65.1)
SWE	63	31	51	10	(45.2–56.7)

The HCPC scored the lowest of all regulators with their document ‘Raise a concern’, scored at 19. The range of lowest scores across the regulators was 19 to 53. A score of 19 means that this can be understood by adults educated to a level of more than 20 years old (tertiary education level), whereas a score of 53 (SCW—How do hearings work) would likely be understood by those with a reading age of 18‐year‐old adults (current school leaver in the United Kingdom). Therefore, all regulators had FtP documents that would be hard to read for most adults in the United Kingdom. The highest scores varied from 44 (PSNI—‘Patient & public guidance’) readable by 19‐year‐old adults to 78 (MPTS—Witness guide to hearings) readable by 11‐ to 12‐year‐old children.

To examine the totality of materials, we applied a mean score to all the general documents. The mean across all documents per regulator in Table 2 shows that all the regulatory bodies' averages are below 64. This means that these materials should be easily understood by 14‐ to 15‐year‐old children. This mean readability score is above the average reading age of adults in the UK. The GMC's tribunal service MPTS has the highest mean score (64%) and PSNI has the lowest (40%). This means that some of the PSNI's materials require the reading equivalent of 17‐ to 18‐year‐old children in the UK, a level attained by only 16% of UK adults. Only NISCC and NMC have narrow gaps between the lower and upper bound confidence intervals (95% confidence level). A narrower gap provides more precise estimation, but it is likely that this precision is affected by the wide range of documents; therefore, the mean values must be interpreted with caution. There exist a number of documents with GMC (*n* = 4), MPTS (*n* = 3) and SCW (*n* = 1) scores ranging from greater than 70 to less than 80, indicating their suitability for readers in years 7 (11–12 years old). These are in accordance with the recommendations of both Health Education England and the American Medical Association [10, 31].

### 3.1 Readability of Easy‐Read Documents

There were 8 easy‐to‐read documents across 4 regulators (Figure 1): 1 each for the GMC, GDC and HCPC and 5 from the NMC. We found that the GMC's document has the highest score, that is, is more readable, as it requires a lower level of reading skill. It scored 88 for the ‘What to do if you are not happy’ document, which means that it should be understandable by adults educated to the level of 10‐ to 11‐year‐old children, which is in line with Health Education England's advice of writing information for patients at the level that can be understood by an 11‐year‐old.

The easy‐read documents of the GDC ‘Report concerns’ and HCPC's ‘How to make a complaint’ both scored 71. This score indicates that adults educated to the level of 14‐ to 15‐year‐old children can easily understand these documents. The NMC has the lowest mean score for their 5 easy‐read documents. Of the 5 NMC documents, ‘How we see’ and ‘The people involved in your case’ scored 59.5 and 59.1. respectively, which means that they are harder to read. However, the NMC document ‘Fitness to Practise—What will happen’ scored 77.2; thus, the readability of this document is 12‐ to 13‐year‐old children, that is, easier to read.

## 4 Discussion

Given that the public are a major source of concerns raised with regulators about health and social care professionals' FtP, it is paramount that the information available can be understood by the breadth of the general adult population. This study found that most documents scored below 80%+, and that overall, the average score for all the regulatory bodies was below 64%, and so, on average, these documents would be difficult to read for at least half of the UK adult population based on the current literacy level in the United Kingodm [30]. In relation to easy‐read documents, only one easy‐read document published by the GMC can be read by the average UK adult, that is, with a literacy level of 9 years. Although other easy‐read documents do not meet the standard, three easy‐read documents by the GDC, HCPC and NMC are not far from this benchmark, but 43% of working‐age adults in the United Kingdom would probably be unable to understand these documents [30]. The result is concerning because when information is too complicated to be read and understood, this may deter some people from raising concerns about registered professionals.

Furthermore, this study suggests that inconsistent readability scores within and across regulatory bodies may make it difficult for people to understand materials fully; they may feel confused and excluded, leading to misunderstanding important parts of the process and reducing trust in regulation. Our inquiries with the regulators revealed that none of the regulators sufficiently prioritised producing materials relating to FtP based on a target readability score. However, this is critical because Subramanian et al. [37] has found that inconsistent readability and quality levels can make people confused about how to navigate information regarding health‐related websites. Our results suggest that most adults will struggle to read the information provided by regulators about FtP, which means that some individuals may find it difficult to follow the process to raise concerns, or they may disengage with the investigation and hearing if they find it difficult to understand the written information about these processes. This may adversely affect the viability of the regulators' case, potentially leading to unsafe practice continuing.

Effective communication of the purposes and processes of FtP, including what information they need to provide and when, requires a consistent approach across all materials. In this sense, readability is important, as it is seen as an effective starting point for determining whether the contents are straightforward and sufficiently well‐written to suit the demands of most people in a country. It has been shown in studies of health literacy that low readability scores indicate that many patients cannot understand the material that they need. This poses risks that they may not proceed with important medical procedures, which may impact patient safety. A lack of easily accessible health information could lead to fear and misunderstanding, affecting health outcomes and participation in preventive care [16]. Therefore, improving readability is recommended by several studies as crucial for enhancing health literacy and patient engagement in their care [16, 17, 18, 19, 20, 21, 22, 23, 24]. There may be similar implications to be drawn from our study that there is a need for consideration of readability when creating and disseminating online information to support public engagement in FtP. The poor overall levels of readability of FtP materials online could be argued to compound health and social care inequities by excluding those with low literacy from FtP.

It is established that well‐composed plain language can facilitate the public to understand the matter fully, which consequently helps them to act [38]. Furthermore, individuals' emotional condition may have an undesirable effect on their usual level of functional literacy. According to Garcia et al. [39], when people are in a specific emotional state, such as anxiety or stress, it motivates them to seek out information, but this emotional state can interfere with their visual processing and interpretation of information. Their anxiety and worry are also heightened if they are unable to understand and absorb information. Consequently, they advised making the contents easily readable. As FtP matters are personal and can be about alleged harm experienced during care by registrants, patients and their family members may access this information when they are dealing with a crisis, distressed, annoyed or confused; also, they may find retelling their story when raising a concern and at multiple points in the FtP process distressing [40]. Therefore, it is recommended that all regulatory bodies achieve at least 70% readability scores of all FtP documents aimed at the public. We also found that employers of registrants thought that the information for registrants as well as their patients and service users should be more readable and inclusive [41]. Further, as 16% of adults in England have very low proficiency in literacy, which is at or below the literacy levels of 5‐ to 7‐year‐old school children [42], and 61% of working‐age adults find it difficult to understand health and well‐being information in England [7], other means of communication, such as the use of videos, are recommended.

## 5 Limitations

What is measured varies by formulas, but most often, they measure certain attributes of words and sentences in isolation. It needs to be noted that the results of these formulas assume that longer words and sentences are hard to understand, whereas short sentences are easy to read. They fail to take familiarisation of words, style, syntax, cohesion and graphics into consideration; all these factors matter with the length of words and sentences, to fully appreciate the readability of a written text [3, 39]. Readability tests should not be accepted as a definitive measure of the overall suitability of documents.

All the measures rely on school grade‐level scores, which can be misleading because high scores provide certainty to people that texts are clear and easy to read when they are may not be in the context of those reading it, for example, if they are not familiar with types of jargon using short words. Nevertheless, all readability formulas are reliable, valid, practicable, cost‐effective, simple, understandable and useful to indicate readability.

The focus of this research was only on readability. Further study could assess the understandability of FtP documents by focusing on word choice, use of visual aids, layout and structure. The Patient Education Materials Assessment Tool (PEMAT) could be used to assess understandability, which is widely used. There are other tools that can be used to analyse the complexity of documents. There is a set of guides on the Plain English website (http://www.plainenglish.co.uk/free-guides.html). There is a tool for detecting the use of length of sentences and overcomplex words (http://www.plainenglish.co.uk/drivel-defence.html). There are common function words that are very frequent in a language, such as it, they, the, but, and, etc. Then, there is a set of about 1000 words that are really common ‘content’ words—the ones that an adult being taught a second language class would use in everyday life: car, plane, dog, cat, sun, rain and so forth After that, each frequency band of another 1000 words can be used but will be read as more complex, irrespective of word length [18], see https://www.lextutor.ca/vp/.

## 6 Conclusions

This is the first study of the readability of the information provided for the public to engage with FtP processes that we are aware of globally. By focussing on the 13 UK statutory health and social care professional regulators, we have shown that, despite some attempts at simplifying language using plain English tools and easy‐read materials by some regulators, none of the regulators' total corpus of information for the public would be accessible by the majority of the UK adult population. We recommend the use of readability scores as well as coproduction of the materials with the end users, which may include registrants as well as the public. All health and social care regulators could improve the readability of their online resources relating to FtP processes, because these materials fell short of the average reading ability of British adults. Increasing readability will undoubtedly increase regulatory literacy and may improve the engagement of the public in professional regulation. More research is needed to investigate not just the readability but also the comprehension in use of FtP documents developed by health and social care regulators, by co‐producing materials with those in the target audience [43].

## Author Contributions

**Sharif Haider:** conceptualisation, investigation, funding acquisition, writing–original draft, methodology, validation, visualisation, writing–review and editing, software, formal analysis, project administration, data curation, supervision, resources. **Louise M. Wallace:** conceptualisation, investigation, funding acquisition, writing–original draft, methodology, validation, visualisation, writing–review and editing, software, formal analysis, project administration, data curation, supervision, resources.

## Ethics Statement

The Open University Human Research Ethics Committee HREC/4058.

## Consent

Participants provided valid informed consent.

## Conflicts of Interest

Louise Wallace is a Lay Panel member for the General Dental Council and Lay Adjudicator for Social Work England.

## Data Availability

Data from this research are available by contacting the corresponding author.
